# Supplementary material for: The causal relationship between the human gut microbiota and pyogenic arthritis: a Mendelian randomization study
Source: Front Cell Infect Microbiol. 2024 Nov 26;14:1452480. doi: 10.3389/fcimb.2024.1452480 (PMC11629706; doi:10.3389/fcimb.2024.1452480)
Supplement: Supplementary file 1 [file DataSheet1.zip › mendelian test/ebi-a-GCST90027764.csv_forest.pdf]

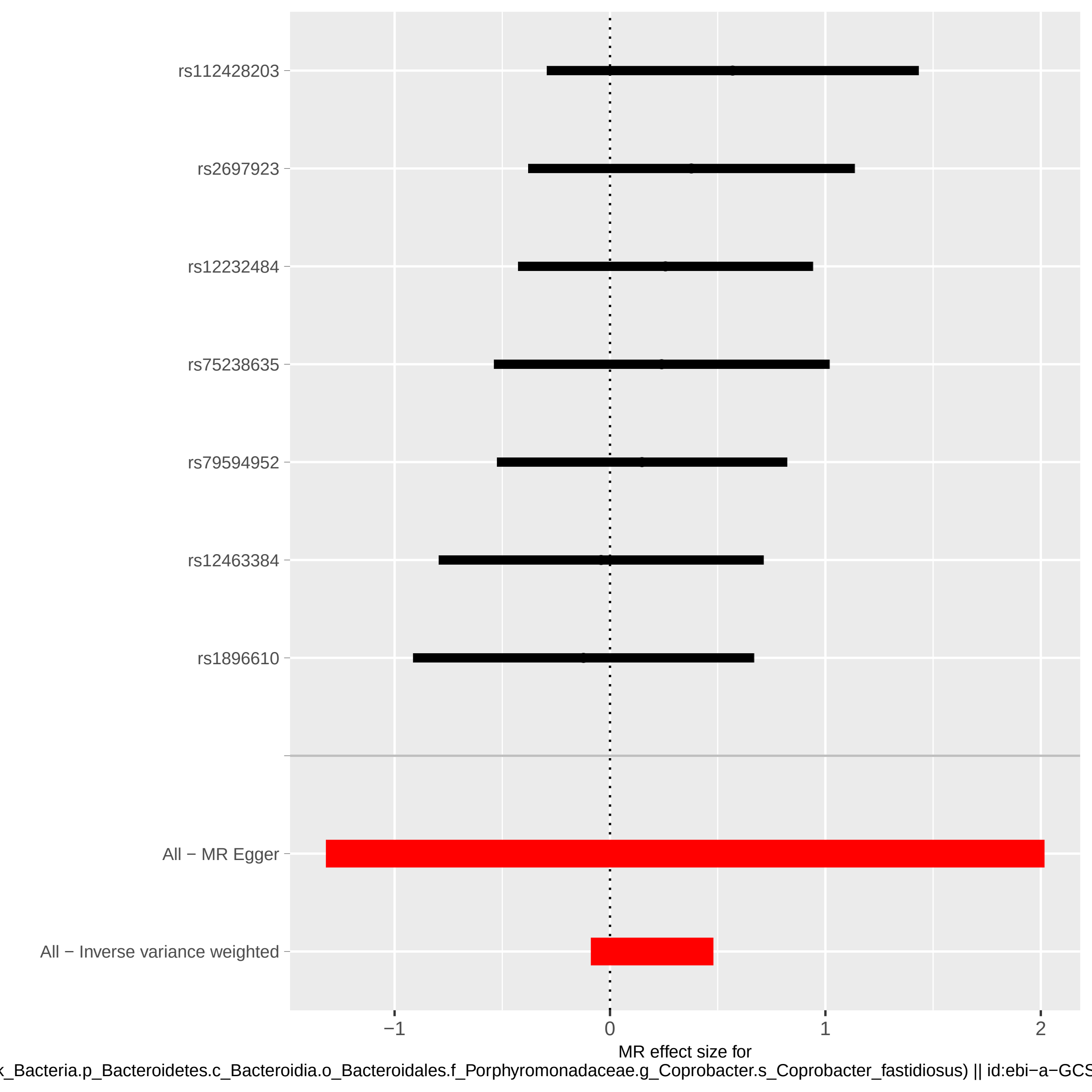

rs112428203
rs2697923
rs12232484
rs75238635
rs79594952
rs12463384
rs1896610
All − MR Egger
All − Inverse variance weighted
−1
0
1
2
MR effect size for
k_Bacteria.p_Bacteroidetes.c_Bacteroidia.o_Bacteroidales.f_Porphyromonadaceae.g_Coprobacter.s_Coprobacter_fastidiosus) || id:ebi−a−GCS
